# Phosphate of Ammonia, in Gout and Rheumatism

**Published:** 1846-03

**Authors:** 

**Affiliations:** Baltimore


*Phosphate of Ammonia in Gout and Rheumatism.* By Dr. Buckler, of Baltimore.—In reviewing the cases which I have published, it will be noticed that thickening in the white tissues, of long standing, has disappeared under the continued use of phosphate of ammonia. Now it is in such cases that the lithic acid diathesis generally prevails, and this agent seems to act here, by depriving the blood for a long time of uric acid and soda, thus creating a demand for these elements in that fluid, and thereby bringing about a re-absorption, as it were, and solution of the superfluous lithate of soda which is deposited in the white tissues. As a result of rheumatism, we sometimes see arthrosis of long standing, where the joints are deformed or dislocated from transformation of tissue, and in gouty subjects we find calcareous deposits, often crippling the joints and interfering with the free play of the tendons. In such cases, we cannot hope for relief in this, or any other agent; but we may relieve the acute attacks which supervene in these chronic cases, and thus save suffering to the patient, and prevent the further increment of calcareous deposit. I have reported only those cases of acute rheumatism in which the phosphate of ammonia was, for the time being, used as a single agent, believing that these would prove more satisfactory to the reader; but I would not be understood as advocating the exclusive use of this remedy; to do this would be the work of an empiric. The leading remedies should be adopted here, as they are in pneumonia, in which disease the lancet is not dispensed with because antimony is used, notwithstanding the efficacy of this last remedy is universally acknowledged.

In acute rheumatism, the disease is seated in the blood, but then there are painful symptoms related with the alteration of this fluid, such as local pain, heat, and swelling in some part of the body, and increased force in the circulation; anodynes will mitigate the one, and the lancet moderate the other, until the primary indication is fulfilled in restoring the blood to its healthy condition. In the case of a boy aged thirteen, who suffered with acute rheumatism in the shoulder and back of the head, and in whose case there seemed to be some implication of the pneumogastric and glossopharyngeal nerves, there being difficulty in deglutition, great aversion to drinks, and irritable stomach, cups were applied to the back of the head, and the following remedies given with the most perfect relief. Tinct. digitalis ʒj; cyanide potass. gr. iij; hyoscyamus ℈ij; phosph. ammonia ʒij; water ℥iv. Two teaspoonfuls every three hours.—*Amer. Jour. of the Med. Sciences.*

